# Bactericidal activity of ceragenin CSA-13, CSA-44 and CSA-131 against bacteria causing bloodstream infections

**DOI:** 10.3389/fmicb.2025.1640392

**Published:** 2025-10-01

**Authors:** Angelika Mańkowska, Paulina Paprocka, Łukasz Suprewicz, Agata Lesiak, Kamila Fortunka, Grzegorz Król, Jakub Spałek, Sławomir Okła, Bonita Durnaś, Tamara Daniluk, Ewelina Piktel, Paul B. Savage, Robert Bucki

**Affiliations:** ^1^Department of Microbiology and Immunology, Institute of Medical Science, Collegium Medicum, Jan Kochanowski University in Kielce, Kielce, Poland; ^2^Department of Medical Microbiology and Nanobiomedical Engineering, Medical University of Białystok, Białystok, Poland; ^3^Institute of Medical Science, Collegium Medicum, Jan Kochanowski University in Kielce, Kielce, Poland; ^4^Department of Otolaryngology, Head and Neck Surgery, Holy-Cross Oncology Center of Kielce, Kielce, Poland; ^5^Independent Laboratory of Nanomedicine, Medical University of Białystok, Białystok, Poland; ^6^Department of Chemistry and Biochemistry, Brigham Young University, Provo, UT, United States

**Keywords:** blood infections, antimicrobial peptides, ceragenins, cytokines, permeability, endothelial cell

## Abstract

**Introduction:**

The constantly growing resistance of bacteria causing bloodstream infections and the lack of alternative antibiotics generate the need to develop new therapeutic strategies. In this study, the antibacterial properties of synthetic cholic acid derivatives ceragenins CSA-13, CSA-44 and CSA-131, custom-synthesized peptides human cathelicidin LL-37 peptide, synthetic WLBU2 peptide, and antimicrobial VFR12 peptide of human thrombin origin were evaluated to determine their potentials as therapeutic agents for bloodstream infections.

**Methods:**

Minimum inhibitory concentrations/minimum bactericidal concentrations (MIC/MBC) against clinical bacterial strains were measured and compared with activity of clinically used antibiotics colistin and vancomycin. Therapeutic potentials of the tested agents were assessed in the presence of 50% blood plasma, and their hemolytic properties were determined using human red blood cells (RCB). Additionally, the antimicrobial activity of CSA-13 against selected clinical strains was assessed using a killing assay. Plasma cytokine levels were determined, and endothelial cell confluent monolayer permeability was assessed using the FITC-dextran and measurement of transepithelial electrical resistance (TEER).

**Results:**

Under experimental conditions mimicking blood environment, ceragenins display higher antimicrobial activity compared to the cationic peptides regardless of the bacterial species. The presence of blood plasma slightly decreases the effect of ceragenins but does not significantly affect their antibacterial properties or their hemolytic activity, especially in case of ceragenin CSA-13. Furthermore, ceragenins at bactericidal concentrations do not induce hemolysis of red blood cells. CSA-13 dose-dependently regulates the permeability of human umbilical vein endothelial cells (HUVECs) monolayers as well as affects the secretion of cytokines, which may indicate its ability to modulate immune responses.

**Conclusion:**

Results presented herein demonstrate the antibacterial activity of ceragenins against clinical strains of bacteria isolated from blood, their influence on the immune system and the integrity of the endothelial cell monolayer. Further studies are necessary to understand the cell signaling pathway governing these effects.

## 1 Introduction

**Figure 1:**
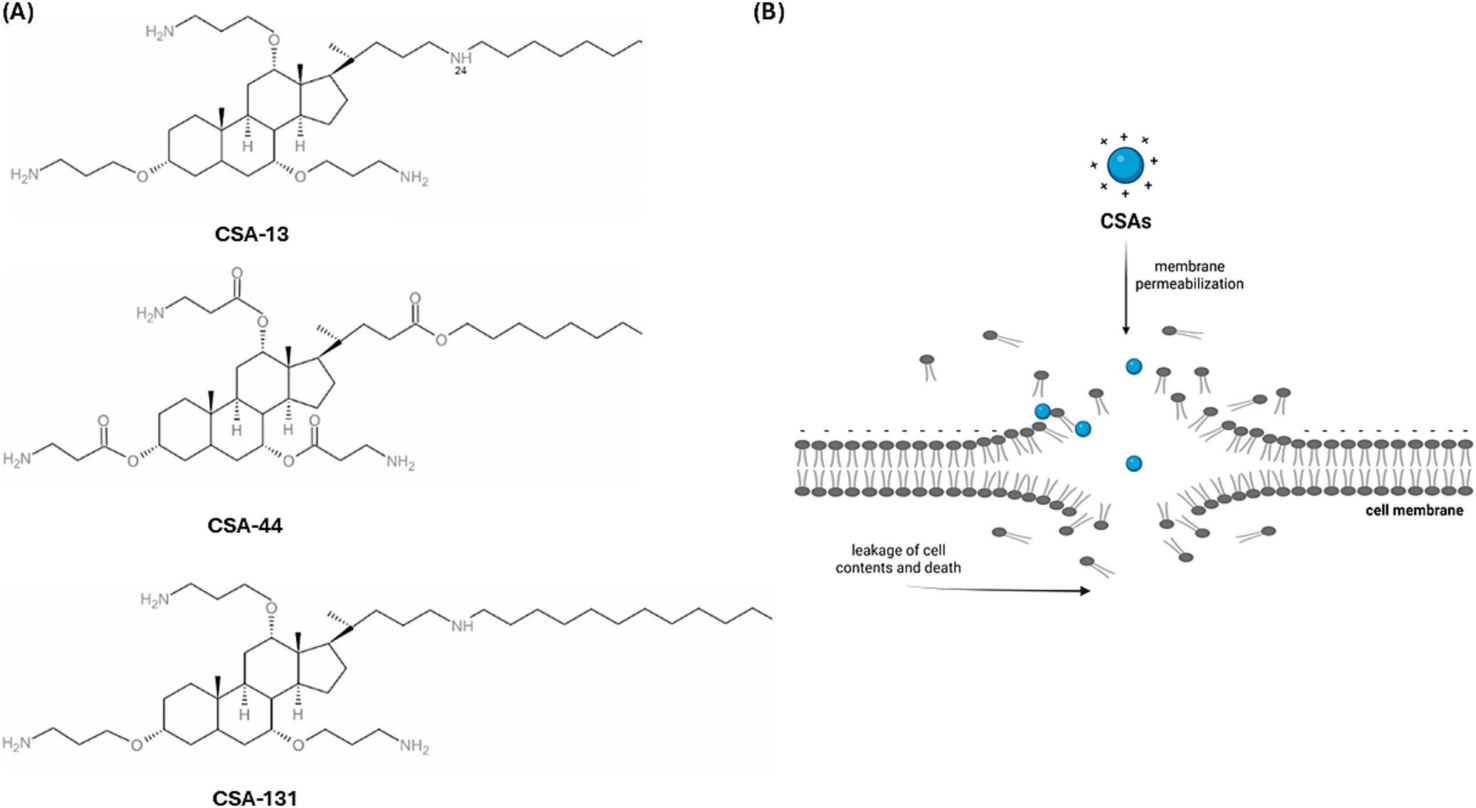
The chemical formula of ceragenins, which are derivatives of cholic acid **(A)** (Karasiński et al., 2024) Schematic representation of the action of ceragenin on the plasma membrane, which results in damage to its continuity **(B)** [Biorender.io software].

Antimicrobial-resistant strains of microorganisms are a major cause of mortality in patients with bloodstream infections. This threat is particularly significant among immunocompromised patients with risk factors, such as damage to mucous membranes and skin, chemotherapy-induced neutropenia, or the use of immunosuppressive drugs in transplant recipients. It is especially concerning in those who have undergone hematopoietic stem cell transplants, as well as in patients with blood cancers or autoimmune disorders (Wang et al., 2024; de Souza et al., 2024; Pezzani et al., 2024). The growing threat of this clinical form of infection requires the search for new therapeutic strategies, especially antimicrobial drugs active against strains of antibiotic-resistant bacteria (Papadimitriou-Olivgeris et al., 2022). A promising experimental direction is the search for new antibiotics that are synthesized using the molecular characteristics of natural antimicrobial peptides that are widely present in nature (Nandi et al., 2022). An example of such molecules is the group of ceragenins (Figure 1A), which are derivatives of cholic acid, characterized by a positive charge and amphipathic nature, which determines their interactions with bacterial membranes and the ability to interfere with membrane structures, resulting in membrane reorganization and leakage (Epand et al., 2008). Ceragenins mimic the membrane-disrupting properties of antimicrobial peptides (Lai et al., 2008). This molecular mechanism of antimicrobial action, based on the physicochemical interaction of molecular charges and membrane insertion (Figure 1B), results in a wide spectrum of antimicrobial activity that covers Gram-positive G (+) and Gram-negative G (−) bacteria, as well as viruses and fungi (Hashemi et al., 2018; Bozkurt-Guzel et al., 2018; Suprewicz et al., 2023). Ceragenins, like natural antimicrobial peptides, have the ability to stimulate the immune system (Suprewicz et al., 2023) and wound healing (Olekson et al., 2017). They also display high activity against bacterial cells surrounded by exopolysaccharides when adopting a biofilm pattern of growth (Wnorowska et al., 2024). The therapeutic potential of ceragenins is also related to the possibility of using metal nanoparticles as carriers for their delivery to sites of infection (Yang et al., 2024; Karasinski et al., 2023; Wnorowska et al., 2020; Hoppens et al., 2014) and control of their toxicity towards host cells using Pluronic F127 (Leszczyńska et al., 2011). They can also serve as molecules that cover the surface of medical devices, preventing colonization and infections associated with their use (Latorre et al., 2021; Mills et al., 2020; Spałek et al., 2021; Zaugg et al., 2023).

A critical element in the pathophysiology of sepsis is endothelial dysfunction, which correlates with disease severity and mortality. The endothelium performs several vital functions in the human body. It constitutes a barrier between blood and tissues (Dolmatova et al., 2021). Increased endothelial permeability accompanies many pathological conditions, including: atherosclerosis, diabetes, hypertension, cancer metastases and sepsis (Harris and Nelson, 2010), where it leads to the inability to maintain the volume of the vascular system, leading to hypoxia and impaired functions of internal organs (Lee and Slutsky, 2010). Here we evaluate the bactericidal activity of ceragenins CSA-13, CSA-44, and CSA-131 against bacteria isolated from the blood of oncological patients in an experimental setting that includes the presence of human blood plasma or whole human blood. This assessment was motivated by previous reports indicating limited activity of antibacterial peptides in the presence of blood, especially blood lipoproteins and divalent cations (Deslouches et al., 2005; Wang et al., 2004; Wang et al., 1998; Vaara, 2009). Since previous studies have shown that a natural antimicrobial peptide, cathelicidin LL-37, regulates the permeability of the endothelium (Byfield et al., 2011), we assessed whether the effect of its synthetic analogue, CSA-13, would be conserved. Restoring vascular integrity using synthetic analogues of natural antimicrobial peptides may constitute a new therapeutic direction in patients with sepsis.

## 2 Materials and methods

### 2.1 Reference strains and clinical isolates

**Figure 2:**
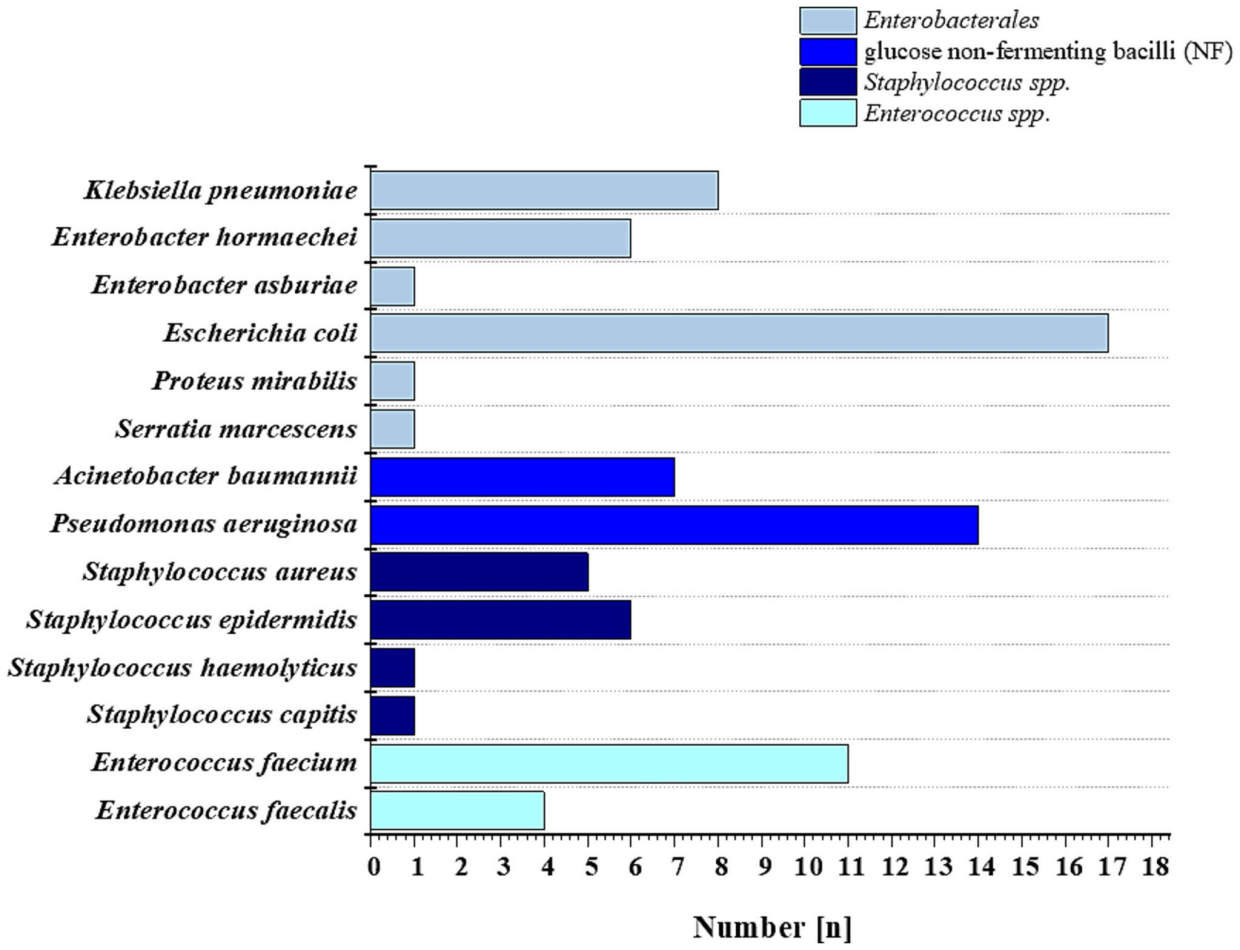
Bacterial strains used in this study were isolated from the blood of patients of the Świętokrzyskie Oncology Center in Kielce, Poland, in 2019–2023.

In accordance with European Committee on Antimicrobial Susceptibility Testing (EUCAST) recommendations (EUCAST, 2023), four reference strains were used for quality control of MIC determinations. This group included: *Escherichia coli* ATCC 25922, *Pseudomonas aeruginosa* ATCC 27853, *Enterococcus faecalis* ATCC 29212, and *Staphylococcus aureus* ATCC 29213 (all purchased from the American Type Culture Collection, Manassas, United States). The study also included 83 clinical strains isolated from the blood of patients hospitalized at the Świętokrzyskie Oncology Center in Kielce, Poland, with clinical symptoms of bloodstream infection. Clinical isolates were obtained from a bank of strains collected between 2019 and 2023 at the Department of Clinical Microbiology of the Świętokrzyskie Oncology Center in Kielce, Poland. Along with the strains, information including the patient’s gender, age, and diagnosis was recorded. The characterization of this collection of strains is presented in Figure 2. The study was approved by the Bioethics Committee of the Collegium Medicum of the Jan Kochanowski University in Kielce (No. 17/2023) and the guidelines contained in the Declaration of Helsinki were followed. The patient’s consent was not necessary because the material used for the research was the leftover material collected during laboratory tests. Before the tests, the samples were plated on ready-made Columbia Agar with sheep blood (Oxoid, United Kingdom) and incubated for 24 h in aerobic conditions at 37 °C. Then, single colonies were identified on a Vitek 2 Compact and Vitek MS Prime MALDI-TOF mass spectrophotometer (bioMérieux, France). Strains for further studies were stored in the MAST CRYOBANK system (Mast Diagnostic, United Kingdom) at −80 °C.

### 2.2 Tested compounds

Ceragenins CSA-13, CSA-44, and CSA-131 were synthesized according to previously described procedures (Ding et al., 2002). Since ceragenins are synthetic analogues of antimicrobial peptides, are positively charged, have an amphipathic nature, and are able of inserting into plasma membrane structures, their activity was compare with that of human cathelicidin LL-37 (LLGDFFRKSKEKIGKEFKRIVQRIKDFLRNLVPRTES), which has an *α*-helical structure containing positively charged amino acids Lys and Arg on the hydrophilic side and several different hydrophobic amino acid residues (Phe, Val, Ile) in the hydrophobic domain (Deslouches et al., 2005; Gudmundsson et al., 1996). The LL-37 peptide is characterized by a broad spectrum of antibacterial activity. Additionally, we decided to evaluate the activity of a synthetic analogue of the LL-37 peptide, namely the WLBU2 peptide (RRWVRRVRRWVRRVVRVVRRWVRR), consisting mainly of Arg (13 residues) and Val (8 residues), with 3 Trp residues on the hydrophobic side, separated by at least 7 amino acids to optimize its activity in blood (Deslouches et al., 2005), and the VFR12 (VFRLKKWIQKVI) peptide, which is derived from thrombin, taking into account the fact that coagulation disorders occur in sepsis (Kasetty et al., 2011) and it can be assumed that its concentration increases with sepsis development. Custom synthesis of the tested peptides was performed at Lipopharm.pl (Zblewo, Poland). Colistin and vancomycin were purchased from Sigma Aldrich (Saint Louis, United States) and Pol-aura (Morąg, Poland) respectfully. Phosphate-buffered saline (PBS) was purchased from Thermo Fisher Scientific, United States.

### 2.3 Assessment of antimicrobial activity

Minimum inhibitory concentrations (MIC) and minimum bactericidal concentrations (MBC) of CSA-13, CSA-44, CSA-131, LL-37, WLBU2, VFR12, colistin, and vancomycin against tested bacterial strains were determined using the serial broth microdilution method in accordance with EUCAST recommendations (EUCAST, 2024). Mueller-Hinton Broth (Oxoid, United Kingdom) or Mueller-Hinton Broth with 50% plasma was used to prepare a series of two-fold dilutions of the appropriate antimicrobial substance, ranging from 0.25 μg/mL to 64 μg/mL. Plasma was obtained by collecting whole blood from healthy, adult donors who gave informed consent (Study was approved by Bioethics Committee of the Collegium Medicum of the Jan Kochanowski University in Kielce – no. 17/2023). Blood was centrifuged at 2,500 g × 10 min and the plasma was collected. A suspension of each strain was then prepared to reach a final concentration of bacteria equal to 5 × 10^5^ CFU/mL. MIC values were taken as the first value at which no turbidity or sediment was observed at the bottom of the well after 18 ± 2 h of bacterial growth. MIC endpoints were assessed visually. To determine MBC, 10 μL of the suspension was taken from the wells corresponding to 1/2MIC, MIC, MICx2 and MICx4, inoculated on LB Agar (BD, Becton Dickinson, Le Pont de Claix, France) and incubated for 24 h at 37 °C. The lowest value at which no growth was observed on solid media was considered the MBC.

### 2.4 Hemolysis measurement

Human red blood cells (RBC) were used to assess the biocompatibility of the tested compounds (CSA-13, CSA-44, CSA-131, LL-37, WLBU2, VFR12, colistin and vancomycin) with respect to host cells. The test was performed in 96-well plates containing RBCs (hematocrit ~ 5%) and tested agents at concentrations ranging from 1 μg/mL to 50 μg/mL. RBCs were suspended in PBS and incubated at 37 °C for 1, 6, and 12 h. After incubation, the plates were centrifuged at 2500 g × 10 min, and the supernatant was transferred to new plates. The amount of hemoglobin released was determined by measuring the absorbance at 540 nm using a Tecan Spark plate reader (Tecan, Männedorf, Switzerland). A sample of RBC containing 1% Triton X-100 was considered as 100% of hemolysis. A value up to 10% is considered non-toxic to the erythrocyte membrane, above 10–49% moderately toxic, 50–89% toxic and 90–100% highly toxic (Pedrozo-Peñafiel et al., 2025). In another set of experiments, the hemolytic activity of CSA-13 was assessed in whole blood. In this setting CSA-13 was tested at 20 μg/mL and 50 μg/mL in 96-well plates and incubated with whole blood. We also assessed if CSA-13 addition affects the response of blood cells to the presence of tested bacterial isolates, including *Pseudomonas aeruginosa*, *Escherichia coli* and *Enterococcus faecium*. The prepared bacterial suspension was added to blood sample at final concentration of 10^5^ CFU/mL and measurements were carried out at time intervals of 1, 3, 6 and 8 h at a temperature of 37 °C. The plates were then centrifuged at 2,500 g for 10 min, the collected sediment was transferred to a sterile plate and the absorbance was measured at 540 nm using a Tecan Spark (Tecan, Männedorf, Switzerland). The positive control was a sample with 1% Triton X-100 (100% hemolysis). This study was conducted as previously described (Chmielewska et al., 2020).

### 2.5 Time-kill kinetics assay in whole blood

A time-kill assay was performed to determine the bactericidal activity of CSA-13. The effect of CSA-13 was also tested in the presence of whole blood against three clinical strains selected from the tested collection: *P. aeruginosa, E. coli* and *E. faecium*. Individual bacterial colonies were suspended at a concentration of 10^8^ CFU/mL and diluted to 10^5^ CFU/mL in PBS or whole blood. Blood was collected in heparin tubes from healthy volunteers. In 96-well plates, CSA-13 was prepared in the concentration range of 0.5–50 μg/mL, and then a previously prepared bacterial suspension was added. Incubations were performed in time intervals: 1, 3, 6 and 8 h at 37 °C. The plates were then placed on ice to inhibit the action of the compounds. Dilutions of 1:10, 1:100, and 1:1,000 were made in PBS, and 10 μL of each sample was inoculated onto LB agar and incubated overnight at 37 °C. Colony-forming units (CFU/mL) were then determined for each sample based on the dilution factor.

### 2.6 Determination of cytokine levels in plasma

Cytokine secretion was assessed using the Human Cytokine Array Kit (Bio-techne, R&D Systems, Minneapolis, MN). Four conditions were prepared on a 96-well plate: whole blood, whole blood + CSA-13 (20 μg/mL), whole blood + CSA-13 (20 μg/mL) + *P. aeruginosa* and whole blood + *P. aeruginosa* and incubated for 6 h at 37 °C. *P. aeruginosa* was suspended in whole blood at 10^5^ CFU/mL. The plates were centrifuged, and the supernatant (plasma) was collected and processed according to the manufacturer’s protocol. Membranes were imaged using a Chemidoc imaging system (Bio-Rad, United States). The image was imported into ImageStudio to quantify the pixel density of each protein on the membrane.

### 2.7 Cell culture

Human umbilical vein endothelial cells (HUVECs) purchased from Sigma Aldrich (Saint Louis, United States) were used for the experiment. They were cultured in an endothelial cell growth medium (#211-500, Cell Applications, United States), supplemented with antibiotics, and maintained at 37 °C in a humidified incubator with 5% CO_2_. After reaching 80–90% confluence, the cells were used for further experiments.

### 2.8 Assessment of endothelial barrier permeability and integrity

**Figure 3:**
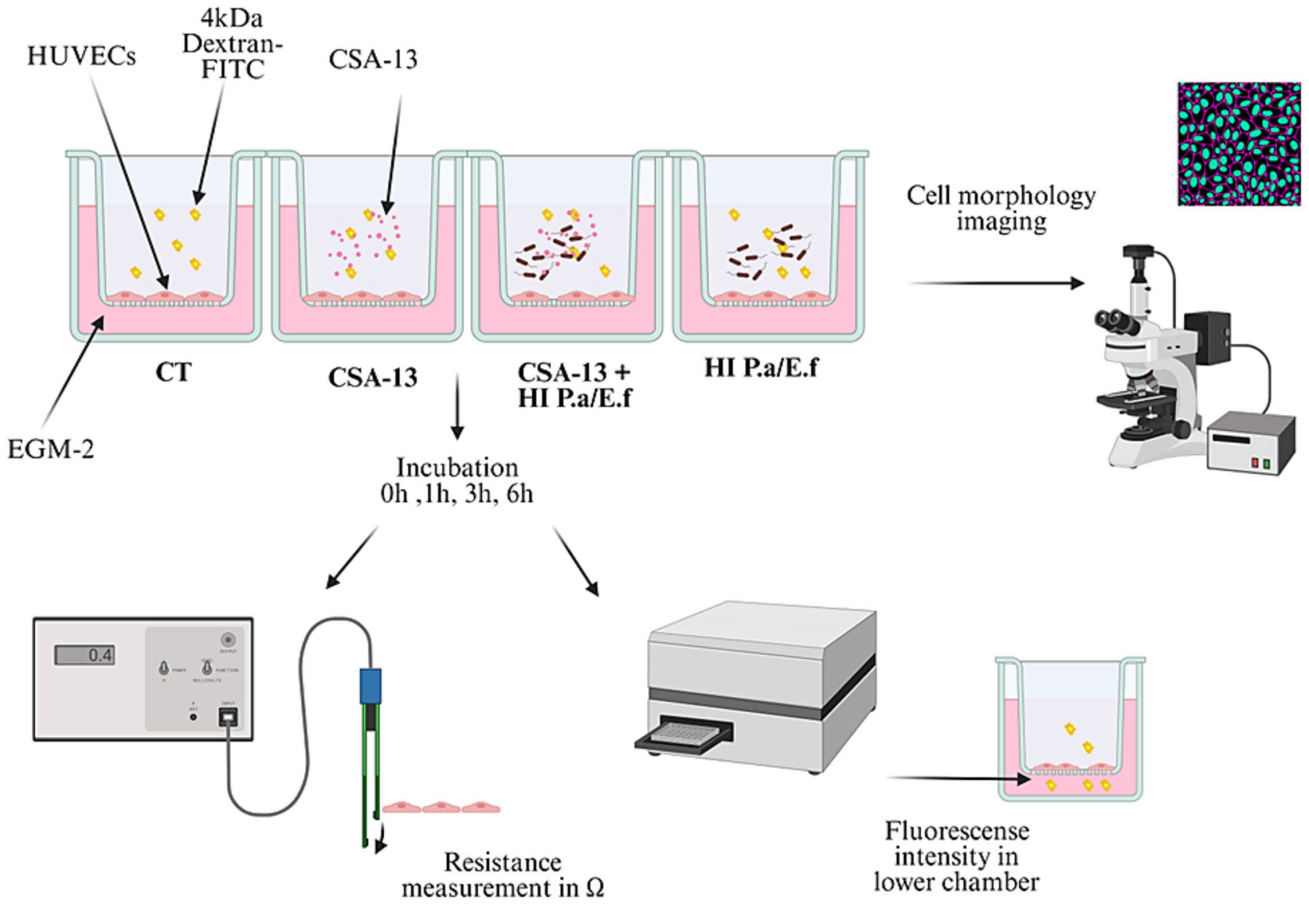
Schematic representation of experimental setting to conduct the permeability/integrity assays using HUVECs monolayers upon addition of ceragenins or their combination with heat-inactivated bacteria [Biorender.io software].

To recreate the functions of the endothelial barrier, cells were seeded at a density of 10^4^ cells per transwell insert coated with collagen I (pore size 0.4 μm, diameter 0.33 cm^2^ #3470, Corning, United States) in 200 μL of complete growth medium. Basolateral chambers were filled with 700 μL of complete growth medium. The medium was changed every 3 days. The formation of the monolayer was confirmed by TEER assessment, reaching maximum, stable values after 5–7 days. After the formation of the monolayer, cells were washed and incubated with CSA-13 at concentrations of 1, 5, and 20 μg/mL, heat-inactivated (autoclaved) bacteria (HI) 10^8^ CFU/mL, and their combinations. Bacterial strains, *P. aeruginosa,* and *E. faecium* were isolated from patients diagnosed with sepsis. A schematic representation of the experimental setup is presented in Figure 3. The barrier integrity measurements were performed before (time 0) and after the addition of the compounds at 1, 3, and 6 h. To evaluate the permeability of the endothelial monolayer, a 4-kDa dextran-FITC (#46944, Sigma Aldrich, United States) was used. The tracer was introduced to the apical chamber of the Transwell at a final concentration of 1 mg/mL. The fluorescent intensity in the lower chamber was tracked using a Varioskan Lux microplate reader (Thermo Fisher Scientific, United States). Percentage permeability was calculated as the relative fluorescence of the medium in treated versus untreated conditions.

At the same time, transendothelial electrical resistance (TEER) was used to assess the permeability of the endothelial barrier. Each sample was measured separately using an EVOM voltmeter (World Precision Instruments, United States) equipped with an STX-2 stick electrode. Barrier resistance readings (Ω) were obtained for each well and, after subtracting the resistance of the blank, were multiplied by the membrane area (0.33 cm^2^) to calculate Ω*cm^2^. The resistance at time 0 for each sample was set to 1.0 to normalize the data. Values of treated samples were normalized to those of an untreated sample (Suprewicz et al., 2022).

### 2.9 VE-cadherin imaging

After 72 h of incubation, treated and untreated sample inserts with HUVECs were washed with PBS and fixed in 4% paraformaldehyde (Sigma Aldrich, United States) for 20 min at room temperature. Cells were permeabilized by adding 0.1% Triton X-100 for 10 min at room temperature and then blocked in 0.3% BSA for 30 min at 37 °C. Samples were then incubated for 48 h at 4 °C with a 1:500 dilution of mouse VE-cadherin monoclonal antibody (Invitrogen, United States). Samples were washed with PBS and incubated with AlexaFluor 647-conjugated secondary antibody (1:1,000) for 1 h in the dark at room temperature. The nuclei were counterstained with Hoechst for 30 min in the dark. Then, the membranes in the cell monolayer were cut out, placed onto a glass coverslip, and mounted with an antifade solution (Abcam, United Kingdom). Fluorescence images were obtained using a Leica microscope DMi8 (Suprewicz et al., 2022).

### 2.10 Statistical analysis

Experiments were performed in 3–6 replicates, values presented are mean ± standard deviation (SD)/standard error (SE). Depending on the type of study, experiments were performed as technical or biological replicates, as described in the figure legends. Significance of differences was determined using a two-tailed Student’s *t*-test with GraphPad software (San Diego, CA) or a one-way ANOVA with Tukey’s *post hoc* test. *p* ≤ 0.05*, *p* < 0.02**, *p* < 0.001 *** were considered statistically significant.

## 3 Results

### 3.1 Antibacterial activity of tested agents

Sensitivity testing of clinical isolates showed that the MIC values of the tested molecules differ depending on the bacterial species. The results are presented in ranges (lower-higher MIC/MBC values) corresponding to species of G (−) and G (+) bacteria (Tables 1, 2, respectively). These data show that in Mueller Hinton Broth (MH) the lowest MIC for *E. coli* was obtained using CSA-13 (0.5–1 μg/mL), for *K. pneumoniae* CSA-131 (1–8 μg/mL), *E. hormaechei* CSA-44 and CSA-131 (1–4 μg/mL), *E. asburiae* CSA-13 (2 μg/mL), *S. marcescens* CSA-13 and CSA-131 (8 μg/mL), *A. baumannii* CSA-131 (1–2 μg/mL) and *P. aeruginosa* CSA-13 and CSA131 (1–2 μg/mL). Interestingly, MICs for CSAs against *P. mirabilis* exceeded > 64 μg/mL, suggesting low susceptibility (natural resistance) of this strain to the tested compounds. In the presence of 50% plasma, CSA-13 retained the highest activity except for activity against *E. asburiae* and *P. mirabilis* (> 64 μg/mL). Generally, in the presence of human plasma, the antibacterial activities of CSA-44 and CSA-131 were decreased compared to their activity in MH media. As for cationic peptides, the lowest MIC values were recorded in the presence of the WLBU2 peptide for most of the above-mentioned species, except *S. marcescens* and *P. mirabilis,* where the MIC was outside the range of tested concentrations > 64 μg/mL. Considering that the MIC values for ceragenins of these two strains were above 64 μg/mL, it is suggested that both strains display lower susceptibility to the tested molecules, whose antibacterial activity requires membrane insertion. This study also confirmed the limited activity of cationic amphipathic peptides in the presence of blood components, particularly for LL-37 and VFR12, where the range of values exceeded 64 μg/mL. Low colistin MIC values were obtained for most of the tested bacterial strains (except for 2 colistin-resistant (R) strains and *Serratia marcescens* and *Proteus mirabilis*, which have natural resistance to colistin) (Aghapour et al., 2019).

**Table 1:** MIC/MBC range for CSA-13, CSA-44, CSA-131, LL-37, WLBU2, VFR12 and colistin in the presence of Mueller Hinton Broth and 50% human blood plasma for the clinical G (−) bacteria strain.

Tested substances	Growth medium	MIC/MBC range μg/mL							
		Organism *n* = 55							
		*Enterobacterales n = 34*						*NF n = 21*	
		*Escherichia colin = 17*	*Klebsiella pneumoniae n = 8*	*Enterobacter* spp. *n = 7*		*Proteus mirabilis n = 1*	*Serratia marcescens n = 1*	*Acinetobacter baumanniin = 7*	*Pseudomonas aeruginosa n = 14*
				*hormaechei n = 6*	*asburiae n = 1*				
CSA-13	Mueller Hinton Broth	0.5–1/0.5–2	4–16/4–16	2–8/2–16	2/4	> 64/> 64	8/8	2–8/2–16	1–2/2–8
	plasma 50%	≤0.25–8/≤0.25–16	1–8/1–16	2–8/2–8	> 64/>64	> 64/> 64	32/>64	32–64/64– > 64	1–8/1–16
CSA-44	Mueller Hinton Broth	1–8/2–16	2–8/2–16	1–4/1–8	16/32	> 64/> 64	32/32	2–4/2–8	2–8/2–16
	plasma 50%	4–32/4–32	8–64/8–64	16–64/16–64	>64/>64	> 64/> 64	> 64/>64	32 – > 64/64– > 64	16–64/32– > 64
CSA-131	Mueller Hinton Broth	0.5–8/0.5–8	1–8/1–16	1–4/1–8	4/4	> 64/> 64	8/8	1–2/1–2	1–2/1–4
	plasma 50%	1–32/1–32	2 – > 64/2– > 64	4–64/4–64	> 64/>64	> 64/> 64	> 64/>64	32 – > 64/64– > 64	4–64/4– > 64
LL-37	Mueller Hinton Broth	32 – > 64/32– > 64	>64/>64	32 – > 64/32– > 64	> 64/>64	> 64/> 64	> 64/>64	16–64/16– > 64	32 – > 64/64– > 64
	plasma 50%	4– > 64/4– > 64	64– > 64/64– > 64	32 – > 64/32– > 64	> 64/>64	> 64/> 64	> 64/>64	> 64/>64	> 64/>64
WLBU2	Mueller Hinton Broth	4–32/4–64	16–64/16–64	4–64/8–64	32/64	> 64/> 64	> 64/>64	8–16/8–16	8–32/8–64
	plasma 50%	2–64/2–64	4– > 64/4– > 64	8– > 64/16– > 64	> 64/>64	> 64/>64	> 64/>64	16–32/32– > 64	4– > 64/4– > 64
VFR12	Mueller Hinton Broth	32 – > 64/64– > 64	> 64/>64	64 – > 64/>64	> 64/>64	> 64/>64	> 64/>64	> 64/>64	16 – > 64/64– > 64
	plasma 50%	0.5 – > 64/0.5– > 64	16 – > 64/16– > 54	32 – > 64/32– > 64	> 64/>64	> 64/>64	> 64/>64	> 64 > 64	16 – > 64/32– > 64
Colistin	Mueller Hinton Broth	≤ 0.25–4/≤0.25–4	≤0.25/≤0.25	≤0.25/≤0.25	> 64/>64	> 64/>64	> 64/>64	≤ 0.25–0.5/≤0.25–2	≤ 0.25–2/≤0.25–4
	plasma 50%	≤ 0.25/≤0.25	≤ 0.25–1/≤0.25–2	≤ 0.25/≤0.25–0.5	> 64/>64	> 64/>64	2/4	≤0.25–1/≤0.25–1	≤ 0.25–0.5/≤0.25–1

**Table 2:** MIC/MBC range for CSA-13, CSA-44, CSA-131, LL-37, WLBU2, VFR12 and vancomycin in the presence of Mueller Hinton Broth and 50% human blood plasma for the clinical G (+) bacteria strain.

Tested substances	Growth medium	MIC/MBC range μg/mL					
		Organism *n* = 28					
		*Staphylococcus* spp. *n = 13*				*Enterococcus* spp. *n = 15*	
		*aureusn = 5*	*epidermidis n = 6*	*capitis n = 1*	*haemolyticus n = 1*	*faecium n = 11*	*faecalisn = 4*
CSA-13	Mueller Hinton Broth	≤ 0.25–2/≤0.25–4	≤0.25/≤0.25	≤0.25/≤0.25	≤0.25/≤0.25	≤ 0.25–1/≤0.25–2	1–2/2–4
	Plasma 50%	4–8/4–8	1–4/1–4	2/2	0.5/1	2–8/2–8	64– > 64/64– > 64
CSA-44	Mueller Hinton Broth	1–2/1–4	0.5–4/0.5–8	2/2	1/2	0.5–2/0.5–4	2–4/2–8
	plasma 50%	32– > 64/32– > 64	16–64/16–64	16/32	16/16	32–64/32–64	64– > 64/64– > 64
CSA-131	Mueller Hinton Broth	0.5–2/0.5–4	≤0.25–2/≤0.25–4	0.5/0.5	0.5/0.5	0.5–1/0.5–2	1–2/2
	plasma 50%	8–64/8– > 64	4–16/4–32	8/8	4/4	16–32/16–32	64– > 64/64– > 64
LL-37	Mueller Hinton Broth	64– > 64/64– > 64	4– > 64/4– > 64	64/64	32/32	32– > 64/64– > 64	32– > 64/>64
	plasma 50%	> 64/>64	64– > 64/64– > 64	> 64/>64	> 64/>64	> 64/>64	> 64/>64
WLBU2	Mueller Hinton Broth	16–64/16– > 64	4–8/4–16	8/8	8/8	2–8/4–16	8–32/16–32
	plasma 50%	32– > 64/32– > 64	4–32/4–32	8/8	8/16	8–64/8–64	> 64/>64
VFR12	Mueller Hinton Broth	> 64/>64	64 – > 64/64– > 64	> 64/>64	64/>64	32 – > 64/64– > 64	> 64/>64
	plasma 50%	> 64/>64	> 64/>64	> 64/>64	> 64/>64	> 64/>64	> 64/>64
Vancomycin	Mueller Hinton Broth	0.5–1/1	1–2/2–4	1/1	2/2	0.5 – > 64/2– > 64	1–4/4–8
	plasma 50%	0.5–2/0.5–4	1–4/2–4	1/1	2/2	0.5 – > 64/0.5– > 64	1–2/1–8

The highest activity against *S. aureus*, *S. epidermidis*, *S. capitis*, and *S. haemolyticus* in MH was observed with CSA-13, giving a range of MIC values of ≤0.25–2 μg/mL, ≤0.25 μg/mL, ≤0.25 μg/mL, and ≤0.25 μg/mL, respectively. In the presence of 50% plasma, the effect of ceragenins decreased; however, CSA-13 had the lowest MIC values among the tested ceragenins and retained its high antibacterial activity. All tested strains of *Staphylococcus* spp. were found to be sensitive to vancomycin, regardless of the type of broth used for MIC determinations. *E. faecium* and *E. faecalis* were the species for which the lowest MICs using CSA-13 and CSA-131 were observed. CSA-13 showed the highest antimicrobial activity in the presence of plasma against *E. faecium*; however, all tested ceragenins partially lost their antimicrobial properties against *E. faecalis* strains in the presence of blood plasma (MIC ranges exceeded > 64 μg/mL). Among the tested peptides against G (+) bacteria, WLBU2 showed the highest antimicrobial activity, at the same time the MIC range for LL-37 and VFR12 exceeded the highest tested concentration. In the case of G (−) bacteria, a similar pattern of activity was observed.

All tested strains of *E. faecalis* were sensitive to vancomycin; however, among the tested strains of *E. faecium*, eight strains were vancomycin-resistant enterococci (VRE). MIC values for reference strains from the ATCC collection were within the range of MIC values for clinical strains. MBCs were determined in the study and the values were close to MIC values (data included in Tables 1, 2).

### 3.2 Hemolysis

**Figure 4:**
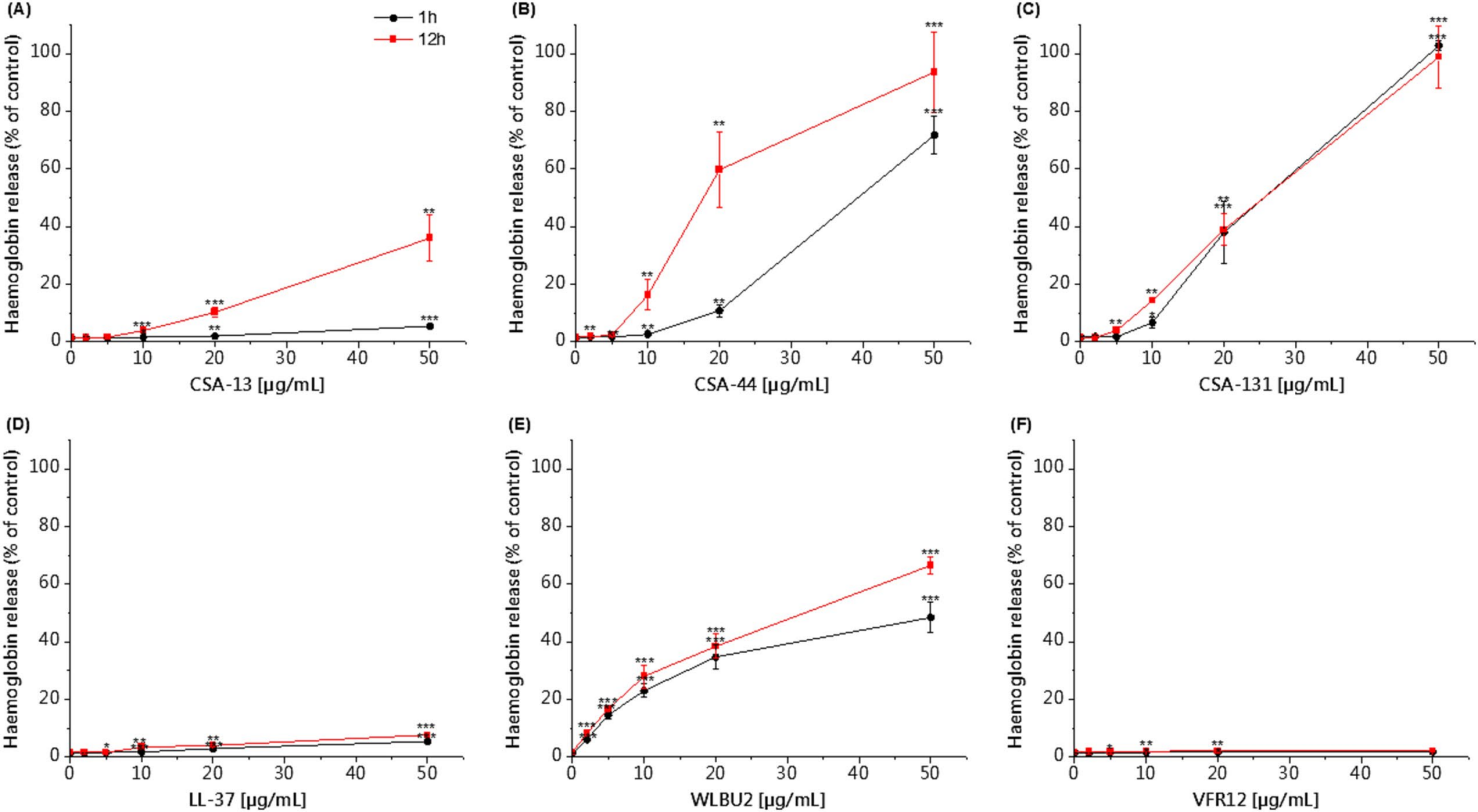
Hemoglobin release from human RBCs after 1 and 12 h of incubation in the presence of CSA-13 **(A)**, CSA-44 **(B)**, and CSA-131 **(C)**, LL-37 **(D)**, WLBU2 **(E)**, VFR12 **(F)**, in the range of 0–50 μg/mL. The results show: mean ± SD, *n* = 3 (biological replicates); * indicates statistical significance compared to the untreated sample, *p* ≤ 0.05, ** < 0.02 and *** < 0.001.

The hemolytic activity of the tested agents was determined using a hemoglobin release assay. Our studies using RBCs showed that CSA-44 and CSA-131 display low hemolysis after 1 h of incubation at a dose of 1–10 μg/mL. After 12 h of incubation, the toxicity of CSA-44 and CSA-131 increased with higher doses ≥10 μg/mL. CSA-13 became slightly toxic above 20 μg/mL. After the application of the WLBU2 peptide, slight toxicity was observed at 5 μg/mL, and this toxicity increased with increasing concentrations of the compound, regardless of the incubation time. LL-37, VFR12, colistin, and vancomycin did not cause significant lysis of human RBCs even at high concentrations (50 μg/mL) after both 1 and 12 h incubation (Figure 4).

CSA-13 showed the strongest antibacterial activity against the tested clinical strains and the highest biocompatibility among the tested compounds. Therefore, it was used to perform additional tests (killing assay, cytokines secretion profile, hemolysis in whole blood and endothelial cell monolayer permeability). From the collection of strains, three clinical isolates were selected for further testing based on their clinical contribution as an important etiological factor of sepsis.

### 3.3 Antimicrobial and hemolytic activity of CSA-13 in the presence of whole blood

**Figure 5:**
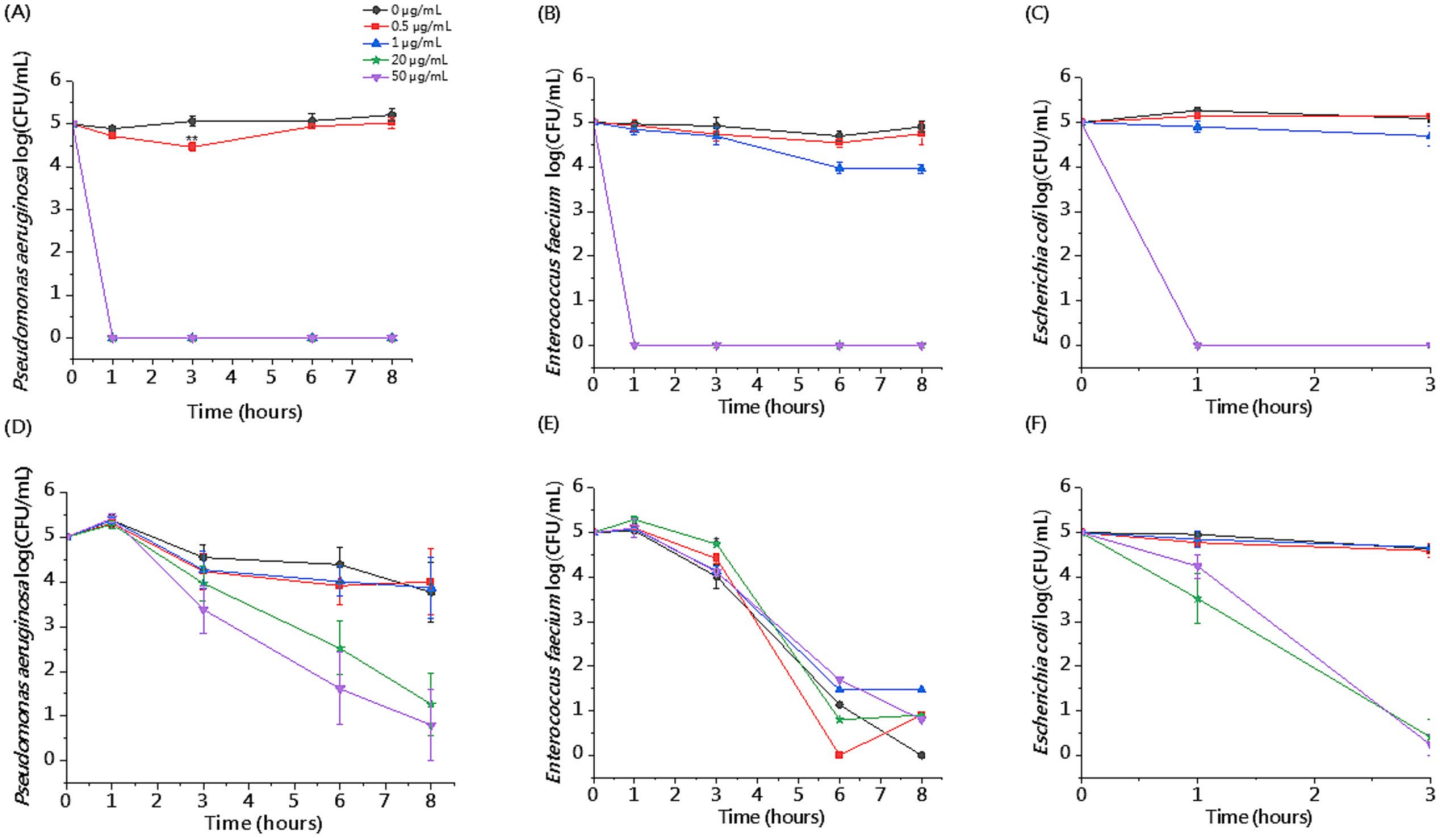
Killing activity of CSA-13 against representative clinical isolates of *P. aeruginosa, E. faecium* and *E. coli* at the dose range of 0.5–50 μg/mL in the presence of PBS **(A–C)** or whole blood **(D–F)**. Results are presented as mean ± SE, *n* = 3–6 (3 biological replicates/6 technical replicates); * indicates statistical significance compared to an untreated sample, *p* ≤ 0.05.

**Figure 6:**
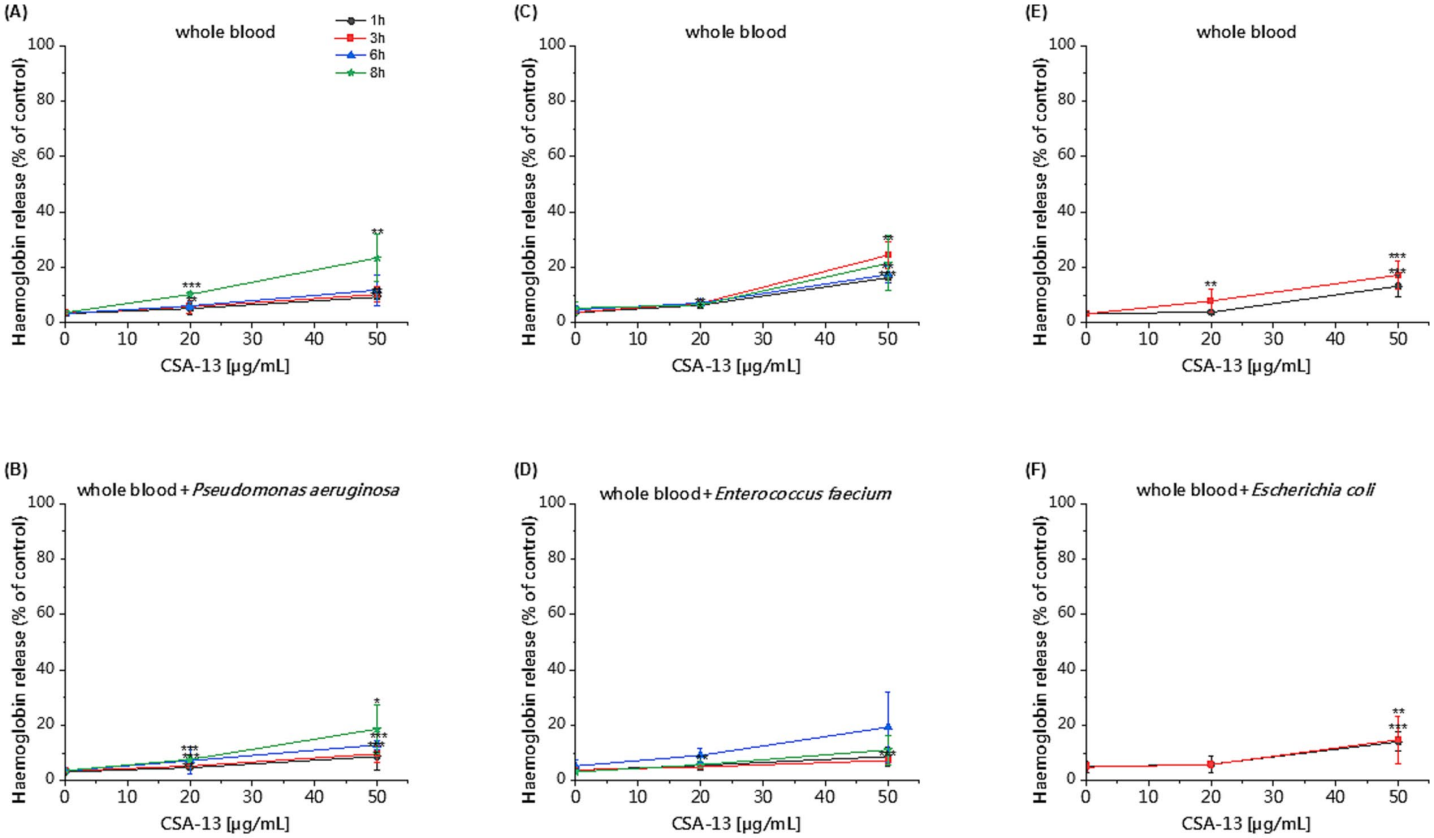
Hemoglobin release after 1, 3, 6 and 8 h of incubation in the presence of CSA-13 at concentrations of 20 and 50 μg/mL in whole blood **(A,C,E)** and whole blood with *P. aeruginosa* **(B)**, *E. faecium* **(D)** and *E. coli* **(F)**. The results show: mean ± SD, *n* = 3–6 (biological replicates); * indicates statistical significance compared to the untreated sample ≤0.05, ** < 0.02 and *** < 0.001.

The killing assay in whole blood enabled us to evaluate the activity of CSA-13 against clinical strains under conditions that mimic the pathophysiological environment developed during sepsis. We observed reduced antibacterial activity of CSA-13 against tested clinical isolates in the presence of whole blood compared to experiments performed in PBS. We recorded the highest activity against *E. coli.* After 3 h, a dose of ≥ 20 μg/mL resulted in growth inhibition. Therefore, we did not perform the determination after 6 and 8 h for this strain. As the dose increased and the incubation time increased, CSA-13 reduced the growth of both *P. aeruginosa* and *E. faecium.* The results are presented in Figure 5. The determination of hemolysis in whole blood in the presence of CSA-13 and three clinical isolates (Figure 6), allowed for the assessment of the safety of ceragenin in an experimental setting simulating blood infection. Our data show that the tested ceragenin, under various experimental conditions, induced hemolysis of some red blood cells. However, this effect was observed at doses that were several times higher than the MIC values. At various time points (1, 3, 6 and 8 h), this effect was observed at 50 μg/mL of CSA-13. In the presence of bacteria, no increased levels of hemolysis were observed compared to untreated samples.

### 3.4 Pro-inflammatory response

**Figure 7:**
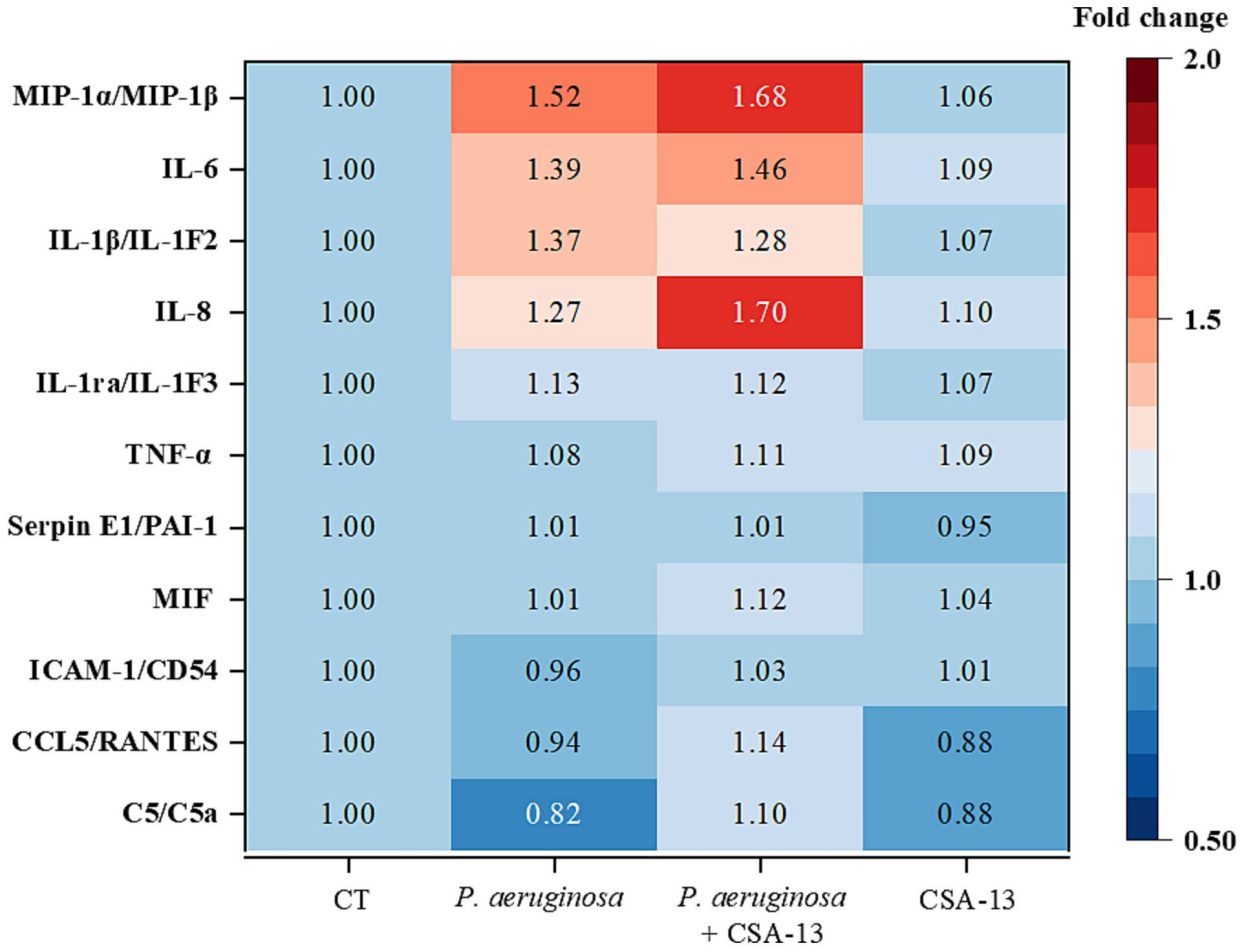
Determination of cytokine levels in plasma after the addition of CSA-13 at a dose of 20 μg/mL (data expressed as fold change compared to the untreated condition, CT). Warmer colors suggest increased cytokine expression, while colder colors suggest decreased expression.

CSA-13 increases the secretion of proinflammatory cytokines, especially interleukin-8 (IL-8), in the presence of *P. aeruginosa* after 6 h of incubation (Figure 7).

### 3.5 CSA-13 reduces bacteria-induced HUVEC monolayer permeability

**Figure 8:**
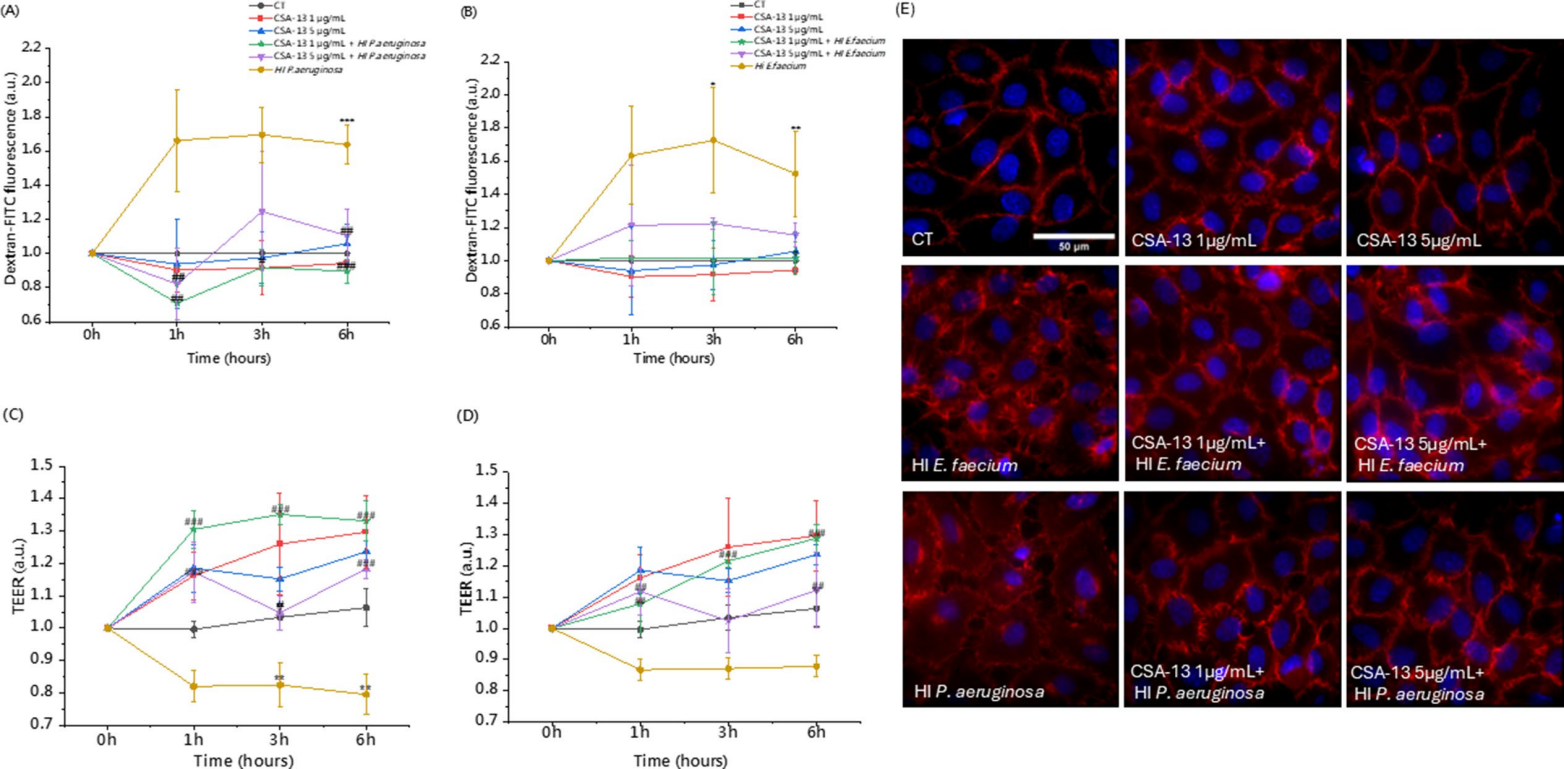
Assessment of the permeability of HUVEC monolayers without and with heat-inactivated bacteria in the presence of CSA-13. Measurements were performed using a fluorometric method based on dextran-FITC permeability **(A,B)** and transendothelial electrical resistance (TEER) **(C,D)**. The results represent the mean ± SD of three measurements (biological replicates), * and # indicate statistical significance at *p* ≤ 0.05, ** /## < 0.02 and *** /### < 0.001 compared to CT and heat-inactivated *P. aeruginosa* **(A,C)**/heat-inactivated *E. faecium* **(B,D)**. VE-cadherin (red) rearrangement in response to CSA-13 with the addition of heat-inactivated *E. faecium* or *P. aeruginosa* **(E)**, nuclei are in blue.

To evaluate the effect of CSA-13 on endothelial cell permeability under various experimental conditions, we performed a dextran permeability assay (Figures 8A,B) and a TEER measurement (Figures 8C,D). Regardless of the time point, heat-inactivated bacteria increased the permeability of HUVEC cells by up to 172% (*Enterococcus faecium* after 3 h). CSA-13 (1 μg/mL and 5 μg/mL) did not induce permeability and a decrease of up to 10% was observed compared to the untreated sample. A dose of 20 μg/mL after 1 h increased the permeability to 156%, but with increasing incubation time, the permeability decreased to 129%. In the presence of heat-inactivated *E. faecium* after 1 h, the permeability reached 163%. After adding CSA-13 at doses of 1, 5, and 20 μg/mL simultaneously with bacteria, the permeability decreased to 101, 121, and 115%, respectively. After 3 h, heat-inactivated *E. faecium* caused an increase to 172%, while 1 μg/mL CSA-13 reduced this effect to 101%, representing a 71% decrease in permeability. After 6 h, heat-inactivated *E. faecium* induced a 152% increase in permeability, while 1 μg/mL CSA-13 induced a 101% increase, representing a 51% decrease in permeability. After 6 h, heat-inactivated *P. aeruginosa* increased permeability to over 160% in 1–6 h. Adding CSA-13 at concentrations of 1 and 5 μg/mL after 1 h reduced permeability to 70 and 82%. A 6 h incubation with CSA-13, 1 μg/mL reduced permeability to 89% and a dose of 5 μg/mL to 110%. CSA-13 regulates the dose-dependent increase in HUVECs permeability induced by bacterial LPS or LTA, with a clear beneficial effect observed at a dose of 1 μg/mL. Measurement of TEER confirms the protective role of CSA-13, which increases the strength of the endothelial cell barrier in a dose-dependent manner. Higher resistance indicates reduced permeability, while lower resistance is associated with impaired permeability. Inactivated bacteria significantly reduced resistance at all time points, while the addition of CSA-13 alleviated the reduction in TEER measurements.

We also evaluated the morphological changes occurring in the monolayer shown in Figure 8E. CSA-13 at doses of 1 and 5 μg/mL in the presence of heat-inactivated *E. faecium* or *P. aeruginosa* does not change the morphology of VE-cadherin intercellular junctions compared to the untreated sample (CT). Delocalization of VE-cadherin from the cell membrane to the cell interior leads to increased permeability resulting from gaps formation between endothelial cells (Lee and Slutsky, 2010). In the presence of inactivated bacteria, delocalization of VE-cadherin was observed. The addition of CSA-13 reduced this effect, which indicates “sealing” of the HUVEC cell monolayer.

## 4 Discussion

Sepsis is caused by a selected population of microorganisms and is accompanied by a complex pathophysiological mechanism (Jarczak et al., 2021). It is a serious clinical problem associated with high mortality, especially among patients hospitalized in intensive care units and with weakened immunity (Sakr et al., 2018). The elimination of pathogens causing sepsis in the hospital environment is often hampered by their emerging resistance to most known classes of antibiotics (Sharma et al., 2024). To improve the outcomes of sepsis treatment, it is necessary to develop new antibacterial drugs with pleiotropic effects (Marques et al., 2023; Vincent, 2022).

Ceragenins have a broad spectrum of antimicrobial activity that includes bacteria, fungi and some viruses (Prasad et al., 2021). Overall, our results are largely consistent with studies previously conducted by Bozkurt-Guzel et al. (2014). Their study assessed, among other things, the activity of CSA-13 against 60 carbapenem-resistant *A. baumannii* strains isolated from the blood of patients with bacteremia. The MIC value for all tested isolates was in the range of 1–16 mg/mL (Bozkurt-Guzel et al., 2014). Another study confirmed the antimicrobial activities of: CSA-8, CSA-13, CSA-44, CSA-90, CSA-131, CSA-138, CSA-142, CSA-144 against MDR Gram (−) bacteria isolated from blood, including *A. baumannii*, *K. pneumoniae*, *E. coli* and *P. aeruginosa* (Yilmaz et al., 2023). Our research confirms the high activity of the tested ceragenins CSA-13, CSA-44 and CSA-131 against G (−) and G (+) bacteria. Comparable to colistin and vancomycin, ceragenins showed higher antimicrobial activity against the tested strains compared to the antimicrobial peptides LL-37, WLBU2, and VFR12. In the study presented here, among the collected clinical isolates, the most numerous were *E. coli n* = 17, *P. aeruginosa n* = 14 and *E. faecium n* = 11. Increasing resistance to colistin, called the “drug of last resort,” has been observed in *A. baumannii* (Qadri et al., 2025), *E. coli* (El-Mokhtar et al., 2021), *P. aeruginosa* (Narimisa et al., 2024), *K. pneumoniae* (Uzairue et al., 2022), as well as the nephrotoxic effect of colistin (Ordooei Javan et al., 2015), or the emergence of strains resistant to vancomycin (Iqbal et al., 2024), constitutes a serious public health problem and the need to search for new medicinal compounds. In this context, it is worth emphasizing our recent animal studies, which showed high activity of ceragenins against *E. coli* causing cystitis in mice, which did not show a toxic effect of ceragenins as a result of histopathological examination of the kidneys in the control group (Wnorowska et al., 2022).

An additional advantage of using ceragenins is their chemical nature, which makes them resistant to the action of proteolytic enzymes, which distinguishes them from natural and synthetic antimicrobial peptides (Czarnowski et al., 2024). Indeed, AMPs such as human cathelicidin (LL-37) may lose their antimicrobial activity when exposed to various proteolytical enzymes in plasma (Yang et al., 2024). However, according to previous reports, plasma might weaken the antibacterial effect of CSA-13 (Leszczyńska et al., 2011) and some antibacterial peptides due to their potential interaction with lipoproteins (Ciornei et al., 2005). In our study we observed high stability of CSA-13 in human plasma compared to CSA-44 and CSA-131 when tested against *E. coli* isolates, where the MIC was in the range of 0.25–8 μg/mL. This range of activity against *E. coli* is in good agreement with previous studies in which uropathogenic *E. coli* bacteria show sensitivity to ceragenin in the range of 1–8 μg/mL (Wnorowska et al., 2022). On this basis, it can be concluded that CSA-13 may be particularly important in the treatment of infections caused by *E. coli*.

In whole blood, the antimicrobial activity of CSA-13 is lower compared to PBS, and a higher dose and longer incubation time are required to observe its effect. We noticed particular bactericidal effectiveness when using CSA-13 at a concentration of ≥ 20 μg/mL against *E. coli* after 3 h of incubation. Blood reduces the pharmacological activity of some amphipathic drugs due to their interaction with blood cells membranes and/or blood plasma components. These findings may open a new avenue of research to understand the pharmacokinetics of ceragenin. Despite these limitations, it is worth noting that CSA-13 at this dose is non-toxic to human erythrocytes and maintains its antimicrobial properties. A limitation of our study was the small group of subjects. In the future, the group of blood donors for whom blood counts will be performed should be increased, as individual differences may affect the obtained results. Individual differences in blood counts impact the risk of infection, the course of disease, and response to treatment. Our study should be considered as a preliminary study evaluating the effects of ceragenin in the presence of whole blood and its components. Additionally, the future, studies using this experimental model should be carried out on a wider group of microorganisms.

Blood contains various immune components cells and molecules, such as white blood cells (WBCs), platelets (PLTs), complement components, and antimicrobial peptides, which have antimicrobial functions (Taha et al., 2019). In our study, *Escherichia coli* grows in the control blood sample, which proves that blood itself does not inhibit the growth of this pathogen. With increasing CSA-13 concentration and increasing incubation time in blood, we observe a decrease in CFU/mL of this bacterium, but we assume that the extension of incubation time may correlate with a reduction in the therapeutic dose of CSA-13.

An alternative to increasing the bioavailability of CSA-13 after intravenous administration may be the use of ceragenin-based nanosystems. According to previous studies, in the presence of body fluids: blood plasma, serum, urine, cerebrospinal fluid, abdominal fluid, sputum, dental plaque (Niemirowicz et al., 2017), ear wax (Prasad et al., 2021), ceragenins and ceragenin-based nanosystems demonstrated strong antimicrobial activity.

An interesting direction of research is the combination of ceragenins with conventional antibiotics in order to reduce the therapeutic dose. Recently, the activity of CSA-44 and CSA-131 against blood clinical isolates of G (−) bacteria in combination with classical antibiotics was reported (Yilmaz et al., 2023). This study highlights that CSA-44 and CSA-131, alone or in combination with meropenem or ceftazidime + avibactam, should be considered when developing new alternative treatments for infections caused by antibiotic-resistant bacterial strains. Another study confirmed the effectiveness of the combination of CSA-13 with colistin, tobramycin and ciprofloxacin (Bozkurt-Guzel et al., 2014).

As a result of infection, the host’s reaction is impaired, which is associated with an excessive pro-inflammatory response (Liu et al., 2022). Therefore, we decided to check whether ceragenins can reduce the inflammatory response. In our previous study, 30 μM CSA-13 and LL-37 reduced IL-8 release by 50% in A549 cells infected with *P. aeruginosa* Xen5 (Bucki et al., 2015). In another study, CSA-13 and CSA-131 effectively reduced the inflammatory response associated with *Gardnerella vaginalis* infection. Reduced levels of MIP-1α/*β*, IL-1α and IL-1β IL-10 was observed (Wnorowska et al., 2024). Our results suggest activation of immune system cells upon blood exposure to CSA-13, as evidenced by the increased levels of MIP-1α and MIP-1β in the CSA-13 + *P. aeruginosa* sample. MIP-1α is an inflammatory protein of macrophages, secreted by cells of the immune system: monocytes, T and B lymphocytes, neutrophils, dendritic cells and NK cells (Bhavsar et al., 2015). In a study by Leszczyńska et al. (2012), CSA-13 was found to induce IL-8 release, which we confirmed here. Proinflammatory cytokine IL-8, has a chemotactic effect on neutrophils and stimulates phagocytosis. Enhanced phagocytosis allows for the restoration of homeostasis which remains disturbed in sepsis (Hortová-Kohoutková et al., 2020). Based on the available experimental data, it can be concluded that CSA-13 has immunomodulatory properties, but further studies are needed to understand its molecular basis. In the future, cytokine levels should be also measured after longer incubation times. It is also worthwhile to test a lower dose of CSA-13.

Endothelial dysfunction, which accompanies sepsis (Joffre et al., 2020), is a crucial determinant of sepsis outcome; therefore, compounds are being sought that will enhance the integrity of this barrier. We used heat-inactivated bacteria to assess endothelial cell permeability. The observed changes are most likely related to immune signaling. In our studies, in addition to its antimicrobial properties, CSA-13 regulates the permeability of endothelial monolayers. We hypothesize that CSA-13 may stabilize the junctions between endothelial cells and V-cadherin and/or modulate inflammatory mediators. Future studies should investigate the mechanism by which CSA regulates endothelial cell permeability. In addition to its antimicrobial properties, CSA-13 regulates the permeability of endothelial monolayers. Another important aspect in the search for new therapeutic agents is the low toxicity of CSAs. The biocompatibility of CSA-13, CSA-44 and CSA-131 has been tested in some previous studies (Paprocka et al., 2022; Skłodowski et al., 2021). Our results confirm low cytotoxicity at bactericidal doses. Toxic doses are several times higher than the MIC values. It is also worth emphasizing that ceragenin-induced host cell damage can be controlled by using Pluronic F-127 (Paprocka et al., 2021), which reduces the toxicity of CSA-13 and does not affect its bactericidal activity (Bucki et al., 2015).

*In vivo,* CSA-13 demonstrates antibacterial efficacy in an animal model of peritoneal infection (Bucki et al., 2015) in a mouse model of urinary tract infections (Wnorowska et al., 2022) in bacterial vaginosis in mice (Wnorowska et al., 2024). To date, there are no data assessing the effect of ceragenin on an animal model of sepsis.

## 5 Conclusion

This study demonstrates the potential of ceragenins, especially CSA-13, as possible agents for developing new therapies for patients with bloodstream infections. Low hemolytic activity at bactericidal concentrations, high antibacterial activity maintained in the presence of plasma and protective function for endothelial cells encourage further evaluation of the potential of this substance in the treatment of sepsis, which is associated with infection and accompanied by endothelial dysfunction. In the future, the focus should be on understanding the molecular mechanism associated with the immunomodulatory function of ceragenins as well as assessing the potential of ceragenins *in vivo* using animal models of sepsis in order to assess the efficacy and safety of these substances from the perspective of potential clinical trials.

## Data Availability

The original contributions presented in the study are included in the article/supplementary material, further inquiries can be directed to the corresponding author.

## References

[ref1] Aghapour Z. Gholizadeh P. Ganbarov K. Bialvaei A. Z. Mahmood S. S. Tanomand A.. (2019). Molecular mechanisms related to colistin resistance in Enterobacteriaceae. Infect. Drug Resist. 12, 965–975. doi: 10.2147/IDR.S199844, PMID: 31190901 PMC6519339

[ref2] Bhavsar I. Miller C. S. Al-Sabbagh M. (2015). “Macrophage inflammatory Protein-1 alpha (MIP-1 alpha)/CCL3: as a biomarker” in General methods in biomarker research and their applications. eds. Preedy V. R. Patel V. B. (Dordrecht: Springer Netherlands), 223–249.

[ref3] Bozkurt-Guzel C. Hacioglu M. Savage P. B. (2018). Investigation of the in vitro antifungal and antibiofilm activities of ceragenins CSA-8, CSA-13, CSA-44, CSA-131, and CSA-138 against Candida species. Diagn. Microbiol. Infect. Dis. 91, 324–330. doi: 10.1016/j.diagmicrobio.2018.03.014, PMID: 29680320

[ref4] Bozkurt-Guzel C. Savage P. B. Akcali A. Ozbek-Celik B. (2014). Potential synergy activity of the novel ceragenin, CSA-13, against carbapenem-resistant *Acinetobacter baumannii* strains isolated from bacteremia patients. Biomed. Res. Int. 2014:710273. doi: 10.1155/2014/710273 24804236 PMC3996866

[ref5] Bucki R. Niemirowicz K. Wnorowska U. Byfield Fitzroy J. Piktel E. Wątek M.. (2015). Bactericidal activity of ceragenin CSA-13 in cell culture and in an animal model of peritoneal infection. Antimicrob. Agents Chemother. 59, 6274–6282. doi: 10.1128/AAC.00653-15 26248361 PMC4576122

[ref6] Byfield F. J. Wen Q. Leszczynska K. Kulakowska A. Namiot Z. Janmey P. A.. (2011). Cathelicidin LL-37 peptide regulates endothelial cell stiffness and endothelial barrier permeability. Am. J. Physiol. Cell Physiol. 300, C105–C112. doi: 10.1152/ajpcell.00158.2010, PMID: 20943960 PMC3023190

[ref7] Chmielewska S. J. Skłodowski K. Piktel E. Suprewicz Ł. Fiedoruk K. Daniluk T.. (2020). NDM-1 Carbapenemase-producing Enterobacteriaceae are highly susceptible to Ceragenins CSA-13, CSA-44, and CSA-131. Infect. Drug Resist. 13, 3277–3294. doi: 10.2147/IDR.S261579, PMID: 33061475 PMC7535143

[ref8] Ciornei C. D. Sigurdardóttir T. Schmidtchen A. Bodelsson M. (2005). Antimicrobial and chemoattractant activity, lipopolysaccharide neutralization, cytotoxicity, and inhibition by serum of analogs of human cathelicidin LL-37. Antimicrob. Agents Chemother. 49, 2845–2850. doi: 10.1128/AAC.49.7.2845-2850.2005, PMID: 15980359 PMC1168709

[ref9] Czarnowski M. Wnorowska U. Łuckiewicz M. Dargiewicz E. Spałek J. Okła S.. (2024). Natural antimicrobial peptides and their synthetic analogues for effective Oral microflora control and Oral infection treatment—the role of Ceragenins in the development of new therapeutic methods. Pharmaceuticals 17:1725. doi: 10.3390/ph17121725, PMID: 39770567 PMC11678171

[ref10] de Souza I. L. A. Cappellano P. Ferreira D. B. Bergamasco M. D. das Chagas Neto T. C. Kerbauy F. R.. (2024). Carbapenem-resistant *Klebsiella pneumoniae* bloodstream infections in haematological malignances and hematopoietic stem cell transplantation: clinical impact of combination therapy in a 10-year Brazilian cohort. PLoS One 19:e0297161. doi: 10.1371/journal.pone.0297161, PMID: 38277372 PMC10817138

[ref11] Deslouches B. Islam K. Craigo J. K. Paranjape S. M. Montelaro R. C. Mietzner T. A. (2005). Activity of the de novo engineered antimicrobial peptide WLBU2 against *Pseudomonas aeruginosa* in human serum and whole blood: implications for systemic applications. Antimicrob. Agents Chemother. 49, 3208–3216. doi: 10.1128/AAC.49.8.3208-3216.2005, PMID: 16048927 PMC1196285

[ref12] Ding B. Guan Q. Walsh J. P. Boswell J. S. Winter T. W. Winter E. S.. (2002). Correlation of the antibacterial activities of cationic peptide antibiotics and cationic steroid antibiotics. J. Med. Chem. 45, 663–669. doi: 10.1021/jm0105070, PMID: 11806717

[ref13] Dolmatova E. V. Wang K. Mandavilli R. Griendling K. K. (2021). The effects of sepsis on endothelium and clinical implications. Cardiovasc. Res. 117, 60–73. doi: 10.1093/cvr/cvaa070, PMID: 32215570 PMC7810126

[ref14] El-Mokhtar M. A. Daef E. Mohamed Hussein A. A. R. Hashem M. K. Hassan H. M. (2021). Emergence of nosocomial pneumonia caused by Colistin-resistant *Escherichia coli* in patients admitted to chest intensive care unit. Antibiotics 10:226. doi: 10.3390/antibiotics10030226, PMID: 33668302 PMC7996192

[ref15] Epand R. M. Epand R. F. Savage P. B. (2008). Ceragenins (cationic steroid compounds), a novel class of antimicrobial agents. Drug News Perspect. 21, 307–311. doi: 10.1358/dnp.2008.21.6.1246829, PMID: 18836587

[ref16] EUCAST. Routine and extended internal quality control for MIC determination and disk diffusion as recommended by EUCAST Version 13.2, valid from 2023-06-29. The European Committee on Antimicrobial Susceptibility Testing (EUCAST). (2023).

[ref17] EUCAST. Reading guide for broth microdilution 2024: Version 5.0. The European Committee on Antimicrobial Susceptibility Testing (EUCAST). (2024).

[ref18] Gudmundsson G. H. Agerberth B. Odeberg J. Bergman T. Olsson B. Salcedo R. (1996). The human gene FALL39 and processing of the cathelin precursor to the antibacterial peptide LL-37 in granulocytes. Eur. J. Biochem. 238, 325–332. doi: 10.1111/j.1432-1033.1996.0325z.x, PMID: 8681941

[ref19] Harris E. S. Nelson W. J. (2010). VE-cadherin: at the front, center, and sides of endothelial cell organization and function. Curr. Opin. Cell Biol. 22, 651–658. doi: 10.1016/j.ceb.2010.07.006, PMID: 20708398 PMC2948582

[ref20] Hashemi M. M. Rovig J. Holden B. S. Taylor M. F. Weber S. Wilson J.. (2018). Ceragenins are active against drug-resistant Candida auris clinical isolates in planktonic and biofilm forms. J. Antimicrob. Chemother. 73, 1537–1545. doi: 10.1093/jac/dky085, PMID: 29635279

[ref21] Hoppens M. A. Wheeler Z. E. Qureshi A. T. Hogan K. Wright A. Stanley G. G.. (2014). Maghemite, silver, ceragenin conjugate particles for selective binding and contrast of bacteria. J. Colloid Interface Sci. 413, 167–174. doi: 10.1016/j.jcis.2013.09.016, PMID: 24183446

[ref22] Hortová-Kohoutková M. Tidu F. De Zuani M. Šrámek V. Helán M. Frič J. (2020). Phagocytosis-inflammation crosstalk in Sepsis: new avenues for therapeutic intervention. Shock 54, 606–614. doi: 10.1097/SHK.0000000000001541, PMID: 32516170 PMC7566305

[ref23] Iqbal F. Alocious A. Joy S. C. Stanly E. A. R. Rajesh V. Unnikrishnan M. K.. (2024). Vancomycin-resistant enterococci: a rising challenge to global health. Clin. Epidemiol. Glob. Health 28:101663. doi: 10.1016/j.cegh.2024.101663

[ref24] Jarczak D. Kluge S. Nierhaus A. (2021). Sepsis—pathophysiology and therapeutic concepts. Front. Med. 8:8. doi: 10.3389/fmed.2021.628302, PMID: 34055825 PMC8160230

[ref25] Joffre J. Hellman J. Ince C. Ait-Oufella H. (2020). Endothelial responses in Sepsis. Am. J. Respir. Crit. Care Med. 202, 361–370. doi: 10.1164/rccm.201910-1911TR, PMID: 32101446

[ref26] Karasinski M. Wnorowska U. Durnas B. Krol G. Daniluk T. Sklodowski K.. (2023). Ceragenins and Ceragenin-based Core-Shell Nanosystems as new antibacterial agents against gram-negative rods causing nosocomial infections. Pathogens 12:1346. doi: 10.3390/pathogens12111346, PMID: 38003809 PMC10674730

[ref001] Karasiński M. Wnorowska U. Daniluk T. Deptuła P. Łuckiewicz M. Paprocka P.. (2024). Investigating the Effectiveness of Ceragenins against Acinetobacter baumannii to Develop New Antimicrobial and Anti-Adhesive Strategies. Int. J. Mol. Sci. 25:7036. Available at: https://www.mdpi.com/1422-0067/25/13/7036 39000144 10.3390/ijms25137036 PMC11241064

[ref27] Kasetty G. Papareddy P. Kalle M. Rydengård V. Mörgelin M. Albiger B.. (2011). Structure-activity studies and therapeutic potential of host defense peptides of human thrombin. Antimicrob. Agents Chemother. 55, 2880–2890. doi: 10.1128/AAC.01515-10, PMID: 21402837 PMC3101415

[ref28] Lai X. Z. Feng Y. Pollard J. Chin J. N. Rybak M. J. Bucki R.. (2008). Ceragenins: cholic acid-based mimics of antimicrobial peptides. Acc. Chem. Res. 41, 1233–1240. doi: 10.1021/ar700270t, PMID: 18616297

[ref29] Latorre M. C. Perez-Granda M. J. Savage P. B. Alonso B. Martin-Rabadan P. Samaniego R.. (2021). Endotracheal tubes coated with a broad-spectrum antibacterial ceragenin reduce bacterial biofilm in an in vitro bench top model. J. Antimicrob. Chemother. 76, 1168–1173. doi: 10.1093/jac/dkab019, PMID: 33544817

[ref30] Lee W. L. Slutsky A. S. (2010). Sepsis and endothelial permeability. N. Engl. J. Med. 363, 689–691. doi: 10.1056/NEJMcibr1007320, PMID: 20818861

[ref31] Leszczyńska K. Namiot D. Byfield F. J. Cruz K. Żendzian-Piotrowska M. Fein D. E.. (2012). Antibacterial activity of the human host defence peptide LL-37 and selected synthetic cationic lipids against bacteria associated with oral and upper respiratory tract infections. J. Antimicrob. Chemother. 68, 610–618. doi: 10.1093/jac/dks434, PMID: 23134677 PMC3566669

[ref32] Leszczyńska K. Namiot A. Cruz K. Byfield F. J. Won E. Mendez G.. (2011). Potential of ceragenin CSA-13 and its mixture with pluronic F-127 as treatment of topical bacterial infections. J. Appl. Microbiol. 110, 229–238. doi: 10.1111/j.1365-2672.2010.04874.x, PMID: 20961363 PMC3386848

[ref33] Liu D. Huang S.-Y. Sun J.-H. Zhang H.-C. Cai Q.-L. Gao C.. (2022). Sepsis-induced immunosuppression: mechanisms, diagnosis and current treatment options. Mil. Med. Res. 9:56. doi: 10.1186/s40779-022-00422-y, PMID: 36209190 PMC9547753

[ref34] Marques A. Torre C. Pinto R. Sepodes B. Rocha J. (2023). Treatment advances in Sepsis and septic shock: modulating pro- and anti-inflammatory mechanisms. J. Clin. Med. 12:2892. doi: 10.3390/jcm12082892, PMID: 37109229 PMC10142733

[ref35] Mills R. J. Boyling A. Cheng T. L. Peacock L. Savage P. B. Tagil M.. (2020). CSA-90 reduces periprosthetic joint infection in a novel rat model challenged with local and systemic *Staphylococcus aureus*. J. Orthop. Res. 38, 2065–2073. doi: 10.1002/jor.24618, PMID: 32009241

[ref36] Nandi A. Yadav R. Singh A. (2022). Phage derived lytic peptides, a secret weapon against *Acinetobacter baumannii*-an in silico approach. Front. Med. (Lausanne) 9:1047752. doi: 10.3389/fmed.2022.1047752, PMID: 36405598 PMC9672511

[ref37] Narimisa N. Keshtkar A. Dadgar-Zankbar L. Bostanghadiri N. Far Y. R. Shahroodian S.. (2024). Prevalence of colistin resistance in clinical isolates of *Pseudomonas aeruginosa*: a systematic review and meta-analysis. Front. Microbiol. 15:1477836. doi: 10.3389/fmicb.2024.1477836, PMID: 39473844 PMC11520190

[ref38] Niemirowicz K. Durnaś B. Tokajuk G. Piktel E. Michalak G. Gu X.. (2017). Formulation and candidacidal activity of magnetic nanoparticles coated with cathelicidin LL-37 and ceragenin CSA-13. Sci. Rep. 7:4610. doi: 10.1038/s41598-017-04653-1, PMID: 28676673 PMC5496903

[ref39] Olekson M. A. You T. Savage P. B. Leung K. P. (2017). Antimicrobial ceragenins inhibit biofilms and affect mammalian cell viability and migration in vitro. FEBS Open Bio 7, 953–967. doi: 10.1002/2211-5463.12235, PMID: 28680809 PMC5494304

[ref40] Ordooei Javan A. Shokouhi S. Sahraei Z. (2015). A review on colistin nephrotoxicity. Eur. J. Clin. Pharmacol. 71, 801–810. doi: 10.1007/s00228-015-1865-4, PMID: 26008213

[ref41] Papadimitriou-Olivgeris M. Jacot D. Guery B. (2022). How to manage *Pseudomonas aeruginosa* infections. Adv. Exp. Med. Biol. 1386, 425–445. doi: 10.1007/978-3-031-08491-1_16 36258082

[ref42] Paprocka P. Durnaś B. Mańkowska A. Skłodowski K. Król G. Zakrzewska M.. (2021). New β-lactam antibiotics and Ceragenins - a study to assess their potential in treatment of infections caused by multidrug-resistant strains of *Pseudomonas aeruginosa*. Infect. Drug Resist. 14, 5681–5698. doi: 10.2147/IDR.S338827, PMID: 34992394 PMC8715797

[ref43] Paprocka P. Mańkowska A. Skłodowski K. Król G. Wollny T. Lesiak A.. (2022). Bactericidal activity of Ceragenin in combination with Ceftazidime, levofloxacin, co-Trimoxazole, and Colistin against the opportunistic pathogen *Stenotrophomonas maltophilia*. Pathogens 11:621. doi: 10.3390/pathogens11060621, PMID: 35745475 PMC9227598

[ref44] Pedrozo-Peñafiel M. Gutierrez-Beleño L. Mendoza C. A. D. Freire-Júnior F. L. Lima M. A. Teixeira T.. (2025). In vitro and ex vivo biocompatibility, biomolecular interactions, and characterization of graphene quantum dots and its glutathione-modified variant for qualitative cell imaging. ACS Omega 10, 16194–16206. doi: 10.1021/acsomega.4c10014, PMID: 40321588 PMC12044483

[ref45] Pezzani M. D. Arieti F. Rajendran N. B. Barana B. Cappelli E. De Rui M. E.. (2024). Frequency of bloodstream infections caused by six key antibiotic-resistant pathogens for prioritization of research and discovery of new therapies in Europe: a systematic review. Clin. Microbiol. Infect. 30, S4–S13. doi: 10.1016/j.cmi.2023.10.019, PMID: 38007387

[ref46] Prasad S. V. Piktel E. Depciuch J. Maximenko A. Suprewicz Ł. Daniluk T.. (2021). Targeting Bacteria causing otitis media using Nanosystems containing nonspherical gold nanoparticles and Ceragenins. Nanomedicine 16, 2657–2678. doi: 10.2217/nnm-2021-0370, PMID: 34823374

[ref47] Qadri M. Tariq H. Mehmood M. S. Saddique M. N. Sajjad W. (2025). Mitigating the global spread of multidrug-resistant *Acinetobacter buamanni* in immunocompromised patients. Discov. Public Health 22:60. doi: 10.1186/s12982-025-00451-7

[ref48] Sakr Y. Jaschinski U. Wittebole X. Szakmany T. Lipman J. Ñamendys-Silva S. A.. (2018). Sepsis in intensive care unit patients: worldwide data from the intensive care over nations audit. Open Forum Infect. Dis. 5:ofy 313. doi: 10.1093/ofid/ofy313, PMID: 30555852 PMC6289022

[ref49] Sharma S. Chauhan A. Ranjan A. Mathkor D. M. Haque S. Ramniwas S.. (2024). Emerging challenges in antimicrobial resistance: implications for pathogenic microorganisms, novel antibiotics, and their impact on sustainability. Front. Microbiol. 15:1403168. doi: 10.3389/fmicb.2024.1403168, PMID: 38741745 PMC11089201

[ref50] Skłodowski K. Chmielewska S. J. Depciuch J. Deptuła P. Piktel E. Daniluk T.. (2021). Ceragenin-coated non-spherical gold nanoparticles as novel Candidacidal agents. Pharmaceutics 13:1940. doi: 10.3390/pharmaceutics13111940, PMID: 34834355 PMC8619546

[ref51] Spałek J. Daniluk T. Godlewski A. Deptuła P. Wnorowska U. Ziembicka D.. (2021). Assessment of Ceragenins in prevention of damage to voice prostheses caused by Candida biofilm formation. Pathogens 10:1371. doi: 10.3390/pathogens10111371, PMID: 34832527 PMC8622639

[ref52] Suprewicz L. Szczepanski A. Lenart M. Piktel E. Fiedoruk K. Barreto-Duran E.. (2023). Ceragenins exhibit antiviral activity against SARS-CoV-2 by increasing the expression and release of type I interferons upon activation of the host's immune response. Antivir. Res. 217:105676. doi: 10.1016/j.antiviral.2023.105676, PMID: 37481038

[ref53] Suprewicz Ł. Tran K. A. Piktel E. Fiedoruk K. Janmey P. A. Galie P. A.. (2022). Recombinant human plasma gelsolin reverses increased permeability of the blood–brain barrier induced by the spike protein of the SARS-CoV-2 virus. J. Neuroinflammation 19:282. doi: 10.1186/s12974-022-02642-4, PMID: 36434734 PMC9694610

[ref54] Taha M. Kyluik-Price D. Kumaran D. Scott M. D. Toyofuku W. Ramirez-Arcos S. (2019). Bacterial survival in whole blood depends on plasma sensitivity and resistance to neutrophil killing. Transfusion 59, 3674–3682. doi: 10.1111/trf.15550, PMID: 31608457

[ref55] Uzairue L. I. Rabaan A. A. Adewumi F. A. Okolie O. J. Folorunso J. B. Bakhrebah M. A.. (2022). Global prevalence of Colistin resistance in *Klebsiella pneumoniae* from bloodstream infection: a systematic review and Meta-analysis. Pathogens 11:1092. doi: 10.3390/pathogens11101092, PMID: 36297149 PMC9607870

[ref56] Vaara M. (2009). New approaches in peptide antibiotics. Curr. Opin. Pharmacol. 9, 571–576. doi: 10.1016/j.coph.2009.08.002, PMID: 19734091

[ref57] Vincent J. L. (2022). Current sepsis therapeutics. EBioMedicine 86:104318. doi: 10.1016/j.ebiom.2022.104318, PMID: 36470828 PMC9782815

[ref58] Wang Y. Agerberth B. Lothgren A. Almstedt A. Johansson J. (1998). Apolipoprotein A-I binds and inhibits the human antibacterial/cytotoxic peptide LL-37. J. Biol. Chem. 273, 33115–33118. doi: 10.1074/jbc.273.50.33115, PMID: 9837875

[ref59] Wang Y. Johansson J. Agerberth B. Jornvall H. Griffiths W. J. (2004). The antimicrobial peptide LL-37 binds to the human plasma protein apolipoprotein A-I. Rapid Commun. Mass Spectrom. 18, 588–589. doi: 10.1002/rcm.1361, PMID: 14978805

[ref60] Wang T. Liu H. Huang H. Weng Y. Wang X. (2024). Colistin monotherapy or combination for the treatment of bloodstream infection caused by *Klebsiella pneumoniae*: a systematic review and meta-analysis. BMC Infect. Dis. 24:161. doi: 10.1186/s12879-024-09024-6, PMID: 38317132 PMC10845734

[ref61] Wnorowska U. Fiedoruk K. Piktel E. Prasad S. V. Sulik M. Janion M.. (2020). Nanoantibiotics containing membrane-active human cathelicidin LL-37 or synthetic ceragenins attached to the surface of magnetic nanoparticles as novel and innovative therapeutic tools: current status and potential future applications. J. Nanobiotechnology 18:3. doi: 10.1186/s12951-019-0566-z, PMID: 31898542 PMC6939332

[ref62] Wnorowska U. Lysik D. Piktel E. Zakrzewska M. Okla S. Lesiak A.. (2024). Ceragenin-mediated disruption of *Pseudomonas aeruginosa* biofilms. PLoS One 19:e0298112. doi: 10.1371/journal.pone.0298112, PMID: 38346040 PMC10861078

[ref63] Wnorowska U. Piktel E. Daniluk T. Paprocka P. Savage P. B. Durnaś B.. (2024). Ceragenins prevent the development of murine vaginal infection caused by *Gardnerella vaginalis*. Pharmaceuticals 17:1445. doi: 10.3390/ph17111445, PMID: 39598357 PMC11597204

[ref64] Wnorowska U. Piktel E. Deptuła P. Wollny T. Król G. Głuszek K.. (2022). Ceragenin CSA-13 displays high antibacterial efficiency in a mouse model of urinary tract infection. Sci. Rep. 12:19164. doi: 10.1038/s41598-022-23281-y, PMID: 36357517 PMC9649698

[ref65] Yang S. Liu F. Leng Y. Zhang M. Zhang L. Wang X.. (2024). Development of Xanthoangelol-derived compounds with membrane-disrupting effects against gram-positive Bacteria. Antibiotics 13:744. doi: 10.3390/antibiotics13080744, PMID: 39200044 PMC11350758

[ref66] Yang Z. R. Qin H. Fan J. W. Du K. Qi L. Hou D.. (2024). Acidity-activated aggregation and accumulation of self-complementary zwitterionic peptide-decorated gold nanoparticles for photothermal biofilm eradication. J. Colloid Interface Sci. 663, 1074–1086. doi: 10.1016/j.jcis.2024.02.018, PMID: 38331692

[ref67] Yilmaz F. N. Öksüz L. Demir E. S. Döşler S. Savage P. B. Güzel Ç. B. (2023). Efficacy of Ceragenins alone and in combinations with antibiotics against multidrug-resistant gram negative pathogens from bloodstream infections. Curr. Microbiol. 80:327. doi: 10.1007/s00284-023-03443-5, PMID: 37620557

[ref68] Zaugg A. Sherren E. Yi R. Larsen T. Dyck B. Stump S.. (2023). Incorporating Ceragenins into coatings protects peripherally inserted central catheter lines against pathogen colonization for multiple weeks. Int. J. Mol. Sci. 24:14923. doi: 10.3390/ijms241914923, PMID: 37834369 PMC10573620

